# Hypoglycemia secondary to factitious hyperinsulinism in a foster care adolescent - a case report of munchausen syndrome in a community hospital emergency department setting

**DOI:** 10.1186/s12873-018-0208-z

**Published:** 2018-12-11

**Authors:** Ashruta Patel, Gary Daniels

**Affiliations:** 1 Philadelphia College of Osteopathic Medicine, Georgia Campus, 625 Old Peachtree Rd NW, Suwanee, GA 30024 USA; 2 Northeast Georgia Medical Center Barrow, 316 N Broad St, Winder, GA 30680 USA

**Keywords:** Factitious disorder, Hypoglycemia, Insulin, Emergency department

## Abstract

**Background:**

Factitious disorder causing hypoglycemia is a psychiatric condition in which patients deliberately use blood sugar lowering medications to cause severe symptoms for the purposes of hospitalization or other primary gains.

**Case presentation:**

We report a case of factitious hypoglycemia in a 19-year-old foster care adolescent female who presented to the Emergency Department with recurrent hypoglycemic episodes, to the degree that the patient required large amounts of dextrose and further management by intensive care unit hospitalization. Further inquiry revealed that the patient intentionally injected herself with large doses of insulin for the purposes of seeking hospital admission.

**Conclusion:**

Factitious disorder in the setting of recurrent hypoglycemia episodes may warrant a psychiatric referral and appropriate discharge follow-up to avoid multiple hospitalizations. Presentation in a non-diabetic patient from insulin use is a type of illness that is a challenge for emergency department physicians to appropriately diagnose and treat. Classic findings include a low blood sugar level, suppressed C-peptide level, and an inappropriately elevated insulin level. Recognizing these psychiatric presentations is crucial in order to stabilize patients and prevent unnecessary testing.

## Background

Factitious disorder (FD) includes motivation that cause individuals to feel a sense of control by sustaining a “sick role” for medical attention and/or emotional support [1, 2]. Motivations for maintaining a “sick role” include: establishing a defined identity, obtaining a more socially acceptable way for psychological or emotional disorders to be taken care of (i.e. though a medical system), hard time dealing with stressors, history of neglect/abuse, addressing resentment towards medical professionals, or carrying out suicidal wishes [1, 3]. FD is rare but could be underdiagnosed or diagnosed as something else and the overall prevalence is estimated to be 0.5–2% [1, 4, 5]. Over 50% of patients have been reported to present with skin injuries or lesions [6]. Risk factors contributing to behaviors associated with factitious disorder include: childhood abuse or neglect; poor parenting; marital issues; substance abuse; and stressful life events [2, 7]. It is possible to have co-existing conditions like, substance abuse, mood disorders, anxiety, personality disorders, and suicidal ideation or history of suicide attempt(s). Health professionals should also consider differential diagnoses, such as malingering, borderline personality disorder, delusional personality, or other psychiatric illnesses. Patients may present with a spectrum of symptoms, medical or psychiatric, common presentations include mirroring symptoms, self-inducing injuries/diseases, presenting incorrect lab reports or false past medical documentation. Early recognition of these symptoms can help prevent extensive work-ups and further harming behaviors. Tactics of fabricating a medical problem include: false electronic medical record histories, mimicking signs or symptoms, interfering with diagnostic testing, physical induction or maintenance of an illness. Patients will present with any symptom [1, 2, 5].

Patients may present with psychiatric symptoms. Psychiatric presentations can further be assessed for symptom validity using psychometric and projective testing [8]. The physical exam could help discover some of the falsifications, such as hidden catheters, foreign bodies, skin lesions inflicted by self [1, 2, 4, 5]. It can be relatively difficult to diagnose factitious disorder with certainty, and all DSM-5 [9] criteria and ICD-10 [10] criteria must be met, respectively: “falsification of physical or psychological signs or symptoms, or induction of injury or disease, associated with identified deception; individual presents himself or herself to others as ill, impaired, or injured; deceptive behavior is evident even in absence of obvious external rewards; behavior is not better explained by another mental disorder, such as delusional disorder or another psychotic disorder” & “persistent pattern of intentional production or feigning of symptoms and/or self-infliction of wounds in order to produce symptoms; no evidence can be found for an external motivation (such as financial compensations, escape from danger, more medical care); absence of a confirmed physical or mental disorder, which could explain the symptoms”.

The symptoms of hypoglycemia indicate an emergency situation because they are due to insufficient glucose delivery to the central nervous system, as glucose is critical and the main source of energy for the brain [11, 12]. Since the brain is only able to produce and store enough glucose, in the form of glycogen, to last a few minutes, a short episode of hypoglycemia can cause mental dysfunction; and, if an episode is severe and/or prolonged, brain damage or death can occur [12].

Here, we present a unique case of serious hypoglycemia requiring admission to the intensive care unit (ICU) in a non-diabetic adolescent female who factitiously injected insulin for the purposes of ED admission and hospitalization.

## Case presentation

A 19-year-old Hispanic female with a past medical history of acne, asthma, and extensive psychosocial distress but no psychiatric diagnoses, presented to the ED with complaints of an episode of lightheadedness, generalized weakness, diaphoresis, diarrhea, and vomiting. Previously, she presented to the hospital with similar complaints two other times; however, she was not seen by our team until the third visit. On this first visit she stated that she used the blood glucose monitor of her girlfriend/roommate, who is a type 1 diabetic, and that her blood sugar reading was 53 mg/dL and later rose to 80 mg/dL after she ate two sandwiches and some chocolate. Upon arriving to the ED, the patient’s symptoms had improved. Vital signs obtained at the time of triage were: blood pressure (BP) 98/65 mmHg, heart rate 81 beats per minute (bpm), respiratory rate 18 breaths per min, oxygen saturation (SpO_2_) of 100% on room air, and an oral temperature of 37 °C (98.6 °F). The patient reported no pertinent past surgical history. She stated that she was allergic to pineapples and that she does not take any medications. In addition, she did not report any tobacco or alcohol use. Initial examination revealed a well-developed, asymptomatic, obese young female in no acute distress. Her blood glucose according to the glucose monitor was 60 mg/dL, which was confirmed with lab draw. Physical exam and labs were unremarkable, and the patient was discharged after being given intravenous (IV) 0.9% sodium chloride (NaCl) for volume restoration, ondansetron for her nausea and vomiting, further directions on diet for hypoglycemia (i.e. adding protein to each meal and eating small frequent meals), and instructions on follow-up with her primary care provider (PCP) for recommendation on further testing for the cause of her hypoglycemic episode and GI symptoms.

The next day, the patient arrived via emergency medical services (EMS) to the ED after she was found unresponsive. She stated that she was not feeling too well due to a virus and became very lightheaded and passed out. Her blood sugar was 46 mg/dL at home prior to administration of half an ampule of dextrose (D50W). Her family mentioned the patient has a history of her blood sugar dropping rather frequently and that they are unsure what to do for this problem. Vital signs obtained at the time of triage were: BP 122/75 mmHg, heart rate 100 bpm, respiratory rate 18 breaths per minute, SpO_2_ of 100% on room air, and an oral temperature of 36.7 °C (98 °F). Physical exam and other labs were unremarkable, and the patient was discharged after being given instructions once again on following-up with a PCP.

Three weeks after her initial presentation, she was rushed to the ED via EMS after being found unresponsive at work with a blood glucose level of 23 mg/dL. She was given 1 ampule of IV D50W by EMS at the scene and her blood sugar rose to 172 mg/dL. Upon arrival to the ED, her blood sugar had dropped back down to 61 mg/dL; and she was noted to again have symptoms of dizziness and decreased alertness with her hypoglycemia. Vital signs obtained at the time of triage were: BP 135/78 mmHg, heart rate 108 bpm, respiratory rate 18 breaths per minute, SpO_2_ of 100% on room air, and an oral temperature of 36.4 °C (97.6 °F). She was given one half of an ampule of D50W IV because of her symptoms of dizziness and evidence of decreased alertness with a decreased blood glucose level, which was suspected to still be dropping. She responded to this treatment with improvement of dizziness and alertness but later had a second hypoglycemic episode in the ED. At this time she was treated with a full ampule of D50W IV and was started on dextrose 5% with 0.45% NaCl (*D5 half*-normal). Despite a brief improvement a few minutes after starting D5 half-normal, she had a third episode of hypoglycemia in the ED and was treated with another ampule of D50W IV and her IV changed to dextrose 10% in water (D10W) at 100 cm^3^ (cc’s) an hour. Despite this change, her blood glucose further dropped to 105 mg/dL from 140 mg/dL, so her D10W was increased to 150 cc’s an hour. Detailed glucose readings throughout this time are reported in Table 1. Her girlfriend/roommate was asked to leave the patient to rest, in order to help determine whether the patient, her roommate, or both might be involved in giving insulin to cause the abrupt hypoglycemic episodes. The patient was weak, confused, profusely sweating with chills, short of breath, nauseated, had heart palpitations, and an altered mental status during all of her hypoglycemic episodes. Of note, once alert, the patient told the charge nurse that her PCP had found a mass in her pancreas that was responsible for her hypoglycemic episodes. Due to the critical nature of her recurrent hypoglycemic episodes in the ED, the patient was admitted to the ICU, where she became more responsive. An electrocardiogram demonstrated normal sinus rhythm, normal axis and intervals, and no acute changes in ST-segment or T wave morphology. The laboratory findings from all three ED visits are reported in Table 2.

**Table 1:** Point-of-Care-Testing (POCT) Glucose on third ED visit/ICU admission day (2/12/2018)

Time	16:45	16:50	17:03	17:46	17:54	17:59	18:27	19:12	19:22
mg/dL	< 30	108	115	37	50	144	98	66	84

**Table 2:** Laboratory findings from the three hypoglycemia ED visits

Test (normal range)	Initial ED Presentation (1/22/2018)	Second ED Presentation (1/23/2018)	Third ED Presentation/ICU admission (2/12/2018)
Hemoglobin (12.5-17 g/dL)	12.4^a^	not collected	12.3^a^
Hematocrit (35–49%)	38.6	not collected	38.6
White Blood Cell (WBC) (4.0–10.5 K/uL)	6.5	not collected	5.3
Platelets (140–400 K/uL)	388	not collected	361
Potassium (3.5–5.0 mmol/L)	3.3^a^	3.5	3.2^a^
Sodium (136–145 mmol/L)	136	136	138
Chloride (98–107 mmol/L)	105	107	107
Bicarbonate (22–29 mmol/L)	26	26	28
Blood Urea Nitrogen (7–18 mg/dL)	12	11	13
Creatinine (0.60–1.30 mg/dL)	0.54^a^	0.56^a^	0.58^a^
Glucose (70–110 mg/dL)	59^a^	76	77
Anion Gap (8–16 mmol/L)	5.0^a^	3.0^a^	3.0^a^
Urine Pregnancy Test	negative	not collected	negative

^a^= abnormal readings

Standard drug screen was done to rule out any symptoms from toxicity; however the results were negative. To determine if factitious use of insulin was the cause for the patient’s presentation, further tests were performed: hemoglobin A1C of 5.1%, C-peptide level of 9.9 ng/mL, free insulin level of 370 mIU/L and a total insulin level of 377 mIU/L.

**Fig. 1:**
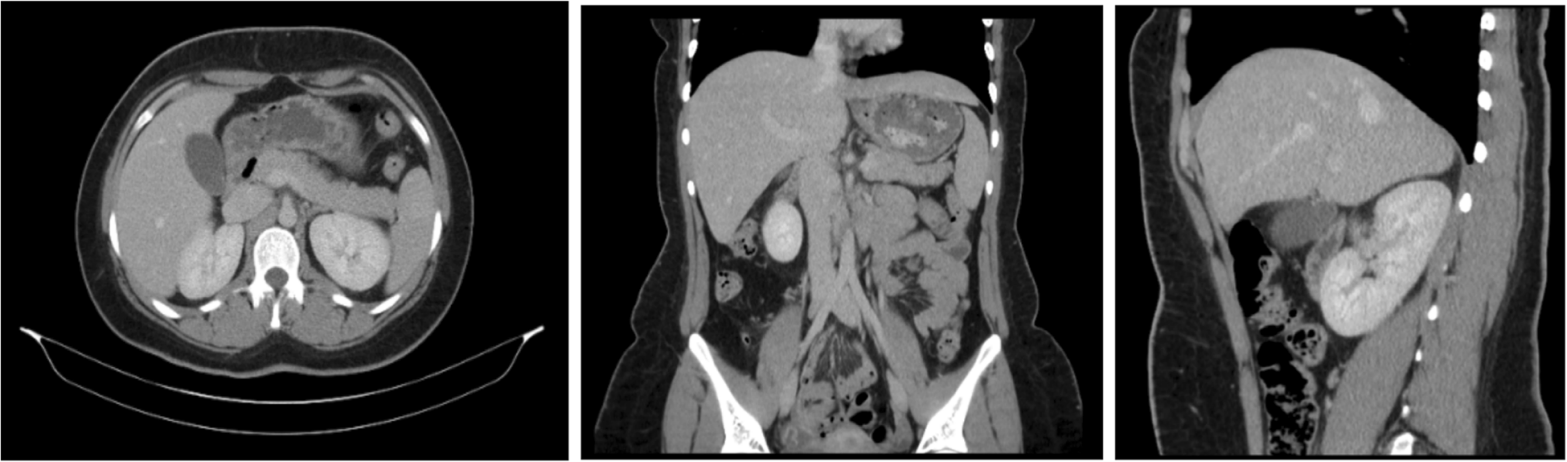
Non-contrast CT images of 19 y/o female in 2018 (**a**) axial (**b**) coronal (**c**) sagittal

To rule out any neurological causes, a computed tomography (CT) scan of the head without IV contrast was performed and revealed no evidence of intracranial findings or suspicious intracranial mass. To rule out any pancreatic masses, CT of the abdomen was obtained and showed a 1.8 cm collapsing cyst in the right ovary and trace fluid in the pelvis, which is likely physiological (Fig. 1). Otherwise, no acute intra-abdominal or intra-pelvic process was seen.

The patient was interviewed at length by the ED hospital team in the ICU the following day, and the diagnosis of factitious disorder was suggested. The encounter revealed that the patient is an undocumented immigrant from Honduras, who has been in the US since she was 14 years old. She used to reside with foster parents. However, she recently graduated from being a minor and is currently living at a halfway house with four other women. She works full-time at a thrift store. She is unaware of her family history. She states that she was heavily involved with illicit drug use in the past but does not smoke or drink alcohol anymore. When confronted with evidence about no mass found on imaging, the patient did not deny injecting insulin. She had significant knowledge about insulin administration as well as the amount of units her girlfriend/roommate currently injects for her type 1 diabetes management. She mentioned that she frequently checks her blood sugar at home, where her readings range from 30s – 100 s mg/dL. The patient reported missing several meals throughout the day due to time restraints and work. Her caregiver called the hospital to let the hospital staff know that the patient had been taking her girlfriend/roommate’s insulin resulting in her previous ED visits. She has also asked for insulin needles and may have taken insulin to gain attention.

## Discussion and conclusions

Factitious disorder imposed on self is a psychiatric illness in which patients create symptoms that are potentially life-threatening for varied reasons. ED physicians encounter multiple psychiatric patients in the emergency room; however, those with factitious disorders are difficult to detect and manage.

There is very little information available on the prevalence of factitious disorder, but it is essential to recognize in an emergent setting. The diagnostic and statistical manual of mental disorders IV (DSM-IV) has three criteria for factitious disorder establishment: 1) deliberate production of physical or psychological signs or symptoms; 2) sick role assumption as motivation; and 3) the absence of external benefits, such as in malingering [9]. The diagnosis is challenging to make in the ED and should be a diagnosis of exclusion [13].

Managing these patients can be a difficult task; and constructive confrontation using a nonjudgmental communicative approach to reduce future incidents could serve as a management goal. It is important to realize when a psychiatric consult would be warranted. Many patients are lost to follow-up after diagnosis, and perhaps involvement of a multidisciplinary team could serve an important role. There are a variety of presentations associated with factitious disorder, and common history elements include: having a previous or current health-related job, diverse symptoms, complex medical history, illness beginning in adolescence, childhood adversity, common coexisting disorders, substance abuse, mood or personality disorders that may not be disclosed, history of factitious disorder in parent, and history of a relative falsifying a disease when they were a child [1, 5, 14]. Psychometric and projective testing involved constructive confrontation, treatment encouragement, non-judgmental discussions about condition, and incorporating a psychiatrist to have appropriate treatment and discharge planning. An interdisciplinary approach may also be beneficial for these patients [1, 2]. In the ED, it may be important to notify and develop a plan for the physician that the patient may return in the next 24 h [15]. There is very limited information available regarding prognosis of factitious disorder, since patients are generally not compliant. However, poor prognosis with a high dropout rate and minimal improvement is reported with any approach, including confrontation or therapy for management of factitious disorder [1, 5, 14, 16].

The suspicion of factitious disorder in our patient occurred after reviewing her three ED visits for hypoglycemic reactions, her relationship with her insulin-dependent diabetic roommate/girlfriend, and her potentially unstable and unhealthy living situation at home. The profound refractory hypoglycemia required a significant amount of dextrose to stabilize, further causing admission to the ICU and monitoring serum glucose levels every 1–2 h. In addition, confirmation of factitious hypoglycemia was made with hemoglobin A1C, C-peptide, and insulin levels. C-peptide is formed form the cleavage of endogenous proinsulin to create insulin. Therefore, administration of exogenous insulin will suppress production of proinsulin and cause a low C-peptide. Our patient’s falsely elevated C-peptide level could be attributed to not fasting prior to the test or insulin resistance. There was no pancreatic mass to suggest an insulinoma on CT of the abdomen. Her creatinine and potassium results were slightly decreased due to her hypovolemic state from diaphoresis and nausea/vomiting; and her serum potassium was also decreased by insulin injection.

Many of these patients are not able to control their self-destructive behavior and require a supportive management approach with urgent psychiatric referral. Psychotherapy has the greatest potential to improve the patient’s condition; but attaining compliance is often difficult. There is one report of successful treatment of factitious disorder in a patient who was committed to working with the mental health staff at the institute for 3 years [17]. Self-handling of insulin as a non-diabetic is a serious condition and may lead to several errors in clinical management for physicians. Research supports the use of validated mental illness screening instruments to help identify psychiatric problems in patients who present as trauma cases [18–20]. Effective screening could further help with institute placement, treatment, and mental health management.

To our knowledge, this is the first reported case of factitious hypoglycemia in the community hospital ED setting in a foster care and halfway house resident adolescent female. This behavior was difficult to predict on initial presentation, especially because the patient did not have any documented psychiatric diagnoses. Therefore, at-risk adolescents should be screened for psychosocial causes even as further critical tests for other hypoglycemia causes are proposed. Factitious disorder is a mental illness in which patients harm themselves for subconscious psychological motives to obtain hospitalization or other primary gains [9, 21]. Factitious hypoglycemia in a non-diabetic patient from insulin use is a type of illness that is a challenge for ED physicians to appropriately diagnose and treat. Classic findings include a low blood sugar level, suppressed C-peptide level, and an inappropriately elevated insulin level. Recognizing these psychiatric presentations is crucial in order to stabilize patients and prevent unnecessary testing.

## Abbreviations

BP: Blood pressure
bpm: Beats per minute
cc’s: Cubic centimeters
CT: Computed tomography
D10W: IV dextrose 10% in water
*D5 half*-normal: Dextrose 5% with 0.45% NaCl
D50W: Dextrose
DSM-IV: Diagnostic and statistical manual of mental disorders IV
ED: Emergency department
EMS: Emergency medical services
NaCl: Sodium chloride
PCP: Primary care provider
POCT: Point-of-care-testing
SpO_2_: Oxygen saturation

## References

[1] Bass C, Halligan P (2014). Factitious disorders and malingering: challenges for clinical assessment and management. Lancet.

[2] Yates GP, Feldman MD (2016). Factitious disorder: a systematic review of 455 cases in the professional literature. Gen Hosp Psychiatry.

[3] Alinejad NA, Oettel DJ (2011). Factitious disorder as repeated diabetic ketoacidosis: a case report. Innov Clin Neurosci.

[4] Kenedi CA, Shirey KG, Hoffa M (2011). Laboratory diagnosis of factitious disorder: a systematic review of tools useful in the diagnosis of Munchausen’s syndrome. N Z Med J.

[5] Kinns H, Housley DFD (2013). Munchausen syndrome and factitious disorder: the role of the laboratory in its detection and diagnosis. Ann Clin Biochem.

[6] Fliege H, Grimm A, Eckhardt-Henn A, Gieler U, Martin K, Klapp BF (2007). Frequency of ICD-10 factitious disorder: survey of senior hospital consultants and physicians in private practice. Psychosomatics.

[7] Beach MC, Gary TL, Price EG (2006). Improving health care quality for racial/ethnic minorities: a systematic review of the best evidence regarding provider and organization interventions. BMC Public Health.

[8] Fontenelle LF, Lins-Martins NM, Melca IA (2014). Exaggerating, mislabeling or simulating obsessive-compulsive symptoms: case reports of patients claiming to have obsessive-compulsive disorder. Compr Psychiatry.

[9] Diagnostic and Statistical Manual of Mental Disorders. 4th ed. Washington, DC; 1994.

[10] Organization WH (1992). The ICD-10 classification of mental and behavioral disorders - clinical descriptions and diagnostic guidelines.

[11] Comi R (1993). Approach to acute hypoglycemia. Endocrinol Met Clin North Am.

[12] Cryer P (1992). Hypoglycemia of obscure case. Hosp Pr.

[13] Bretz S, Richards J (2000). MUNCHAUSEN SYNDROME PRESENTING ACUTELY IN THE EMERGENCY DEPARTMENT. J Emerg Med.

[14] Fischer CA, Beckson MDP (2017). Factitious disorder in a patient claiming to be a sexually sadistic serial killer. J Forensic Sci.

[15] Taylor JB, Beach SR, Kontos N (2017). The therapeutic discharge: an approach to dealing with deceptive patients. Gen Hosp Psychiatry.

[16] Eastwood S, Bisson JI (2008). Management of factitious disorders: a systematic review. Psychother Psychosom.

[17] Yassa R (1978). Munchausen syndrome: a successfully treated case. Psychosomatics.

[18] Dicker RA, Mah J, Lopez D (2011). Screening for mental illness in a trauma center: rooting out a risk factor for unintentional injury. J Trauma - Inj Infect Crit Care.

[19] Joska J, Flisher AJ (2005). The assessment of need for mental health services. Soc Psychiatry Psychiatr Epidemiol.

[20] Macpherson R, Haynes R, Summerfield L, Foy C, Slade M (2003). From research to practice - A local mental health services needs assessment. Soc Psychiatry Psychiatr Epidemiol.

[21] Asher R (1951). MUNCHAUSEN’S SYNDROME. Lancet.

